# Massive Pulmonary Embolism and Deep Vein Thrombosis in COVID-19 Pneumonia: Two Case Reports

**DOI:** 10.7759/cureus.14833

**Published:** 2021-05-04

**Authors:** Siddharth Chopra, Jasmeet Kaur, Mehrvaan Kaur

**Affiliations:** 1 Internal Medicine, St. Joseph Mercy Oakland Hospital, Pontiac, USA; 2 Diagnostic Radiology, St. Joseph Mercy Oakland Hospital, Pontiac, USA

**Keywords:** pulmonary embolism, deep vein thrombosis (dvt), covid-19 pneumonia, covid- 19

## Abstract

Coronavirus disease 2019 (COVID-19) is known to cause a severe acute respiratory syndrome with increased morbidity and mortality due to multiorgan involvement. COVID-19 is associated with an increased risk of venous thromboembolism (VTE), ranging from asymptomatic to potentially fatal presentations. Predictors of VTE in COVID-19 are not fully defined, and the role of anticoagulation in these patients is debatable. Here we discuss two cases of COVID-19, who initially presented with mild COVID-19 symptoms and later with potentially fatal VTE within 30 days of initial presentation. The first case is of a 42-year-old gentleman with a history of sarcoidosis and a recent diagnosis of COVID-19 pneumonia who was in isolation at home and presented with syncope and worsening shortness of breath. He was hemodynamically unstable and resuscitated with fluid management in the emergency department. The chest angiogram imaging studies showed massive pulmonary embolism with right heart strain, which was confirmed with bedside point-of-care ultrasound. The patient deteriorated clinically and received an intravenous tissue plasminogen activator in the emergency. He was discharged home under stable condition on oral anticoagulation. The second patient is a 63-year-old gentleman with chronic obstructive pulmonary disease, obesity, sleep apnea, and a recent diagnosis of COVID-19 pneumonia, which was complicated with an ischemic stroke, who presented with worsening complaints of shortness of breath and palpitation. The chest angiogram imaging showed bilateral pulmonary embolism. An echocardiogram showed mild right heart strain. The lower extremity duplex ultrasound showed bilateral deep vein thrombosis. The patient underwent catheter-directed thrombolysis and discharged on oral anticoagulation. There is a need to develop stronger predictors to provide thromboprophylaxis in COVID-19 pneumonia to prevent life-threatening VTE.

## Introduction

Coronavirus disease 2019 (COVID-19) is known to cause severe acute respiratory syndrome and is accountable for the pandemic leading to approximately 111 million cases and two million deaths worldwide as of February 2021 [1]. Death in COVID-19 patients is reported to be due to severe pneumonia leading to alveolar damage, cardiovascular complications, and kidney failure [2]. The COVID-19 infection has been associated with cytokine storms and hypercoagulable states [2]. Several cases of venous thromboembolism (VTE), including deep venous thrombosis (DVT) and pulmonary embolism (PE), have been reported in the COVID-19 patients [3]. Predictors of VTE in COVID-19 are not fully understood. The role of anticoagulation therapy is under debate. VTE is a morbidity that is a preventable and treatable condition with anticoagulation if the appropriate diagnosis is made on time [4]. Clinicians should suspect PE or DVT in a patient with COVID-19 pneumonia on presentation. Here we discuss two cases of COVID-19 pneumonia who initially presented with mild COVID-19 symptoms and later with potentially fatal VTE within 30 days of initial presentation.

## Case presentation

Case 1

 A 42-year-old Caucasian male with a medical history significant for sarcoidosis, well-controlled essential hypertension, and a recent diagnosis of COVID-19 pneumonia (10 days before presentation), who was in home isolation, presented to the emergency department (ED) after a syncopal episode and shortness of breath. On physical examination, the patient was hemodynamically unstable with a heart rate of 150 beats per minute, blood pressure of 56/24 mmHg, and respiratory rate of 51 breaths/minute. His room air oxygen saturation was 96%, and arterial blood gas revealed hypoxemia with a pO_2_ of 68 mmHg and an elevated alveolar-arterial gradient of 47.5 mmHg (expected: 14.5 mmHg). Chest examination revealed decreased breath sounds bilaterally without wheezing or crackles. The rest of the examination was unremarkable. The patient was stabilized with fluid resuscitation and oxygen therapy in the ED.

**Figure 1:**
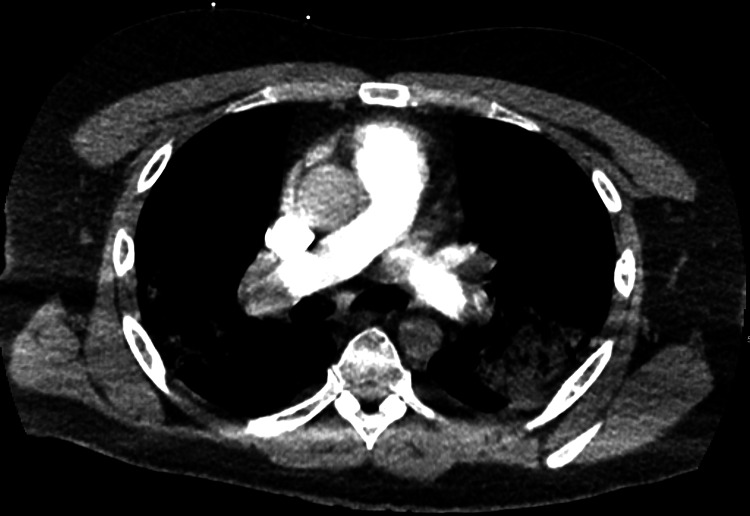
CTA of the chest showing pulmonary embolism. CTA, computed tomography angiogram

**Figure 2:**
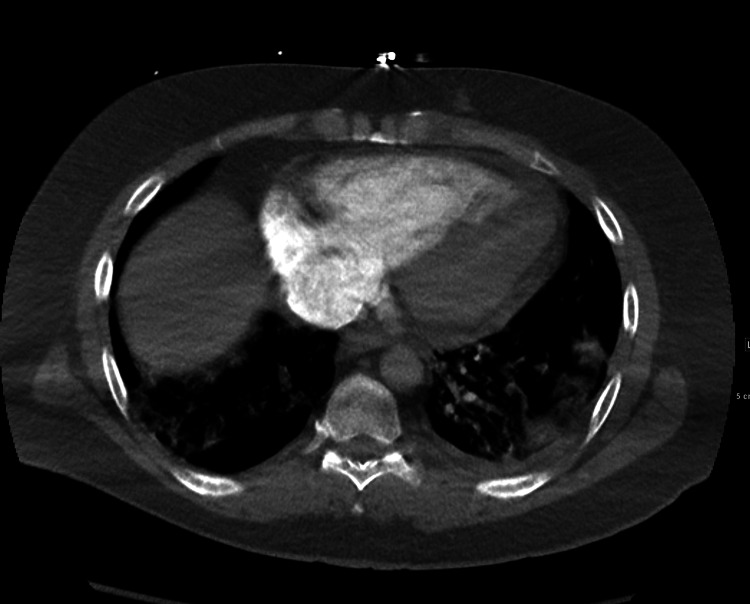
CTA of the chest showing heart strain due to pulmonary embolism. CTA, computed tomography angiogram

**Figure 3:**
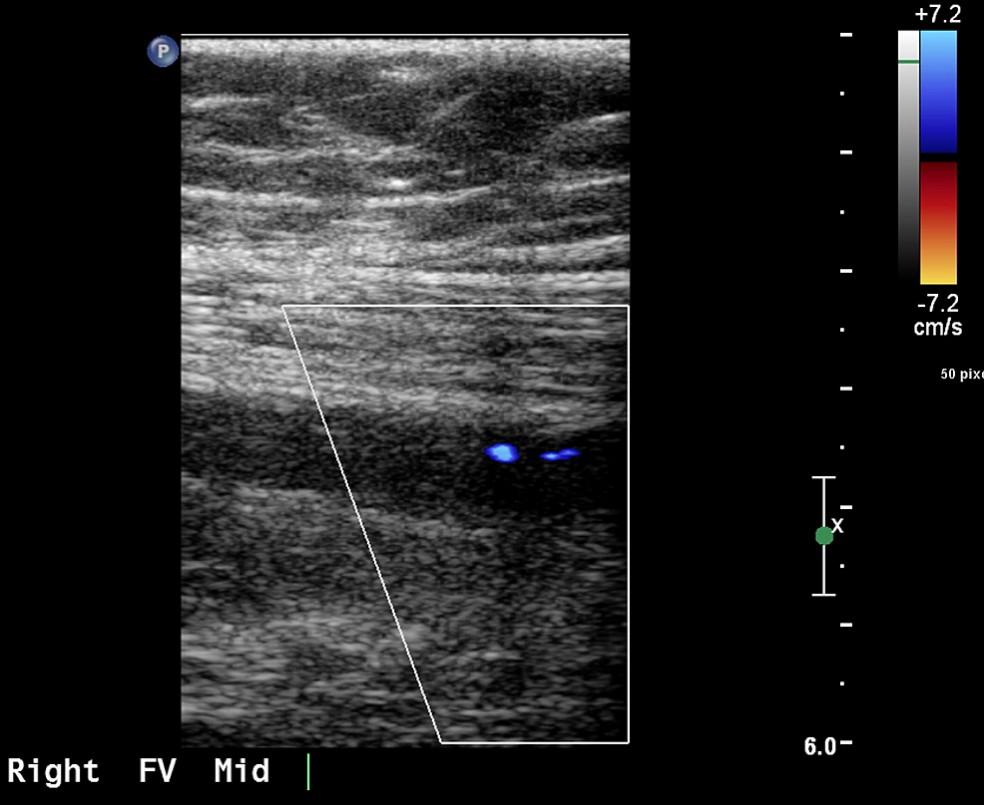
Venous duplex ultrasound of the right lower extremity showing femoral vein thrombosis.

Laboratories workup on presentation is shown in Table 1. Complete blood count revealed an elevated white blood count of 17,600/microliter with absolute neutrophilia. Rapid COVID-19 test was positive. Electrocardiogram (EKG) revealed sinus tachycardia and ST and T wave changes. Computed tomography angiogram (CTA) of the chest revealed acute occlusive and non-occlusive bilateral pulmonary emboli with suspected saddle embolism component, with a large emboli pattern with right heart strain confirmed with point-of-care ultrasound (Figures 1, 2). Computed tomography (CT) of the head was unremarkable. Vascular ultrasound of the bilateral lower extremities revealed acute right extremity DVT extending from the right superficial femoral veins to the calf veins (Figure 3). He was hemodynamically unstable and clinically deteriorating in the ED. Mechanical thrombectomy was not possible. He received tissue plasminogen (tPA) factor in the ED and was started on intravenous (IV) heparin infusion, which was continued over the course of his stay in the hospital. He was clinically improved throughout his stay for five days and was discharged on three months of oral anticoagulants.

**Table 1:** Baseline characteristic and laboratory values. DM; diabetes mellitus; COPD, chronic obstructive pulmonary disease; WBC, white blood count; Hb, hemoglobin; AST, aspartate transaminase; ALT, alanine transaminase; CRP, C-reactive protein; PT, prothrombin time; PTT, partial thromboplastin time; INR, international normalized ratio; LDH, lactate dehydrogenase; BNP, brain natriuretic peptide; BMI, basal metabolic index

	Case 1	Case 2
	Admission	Admission
Age	42 years	63 years
Race	Caucasian	African American
Sex	Male	Male
Hypertension	Yes	No
DM	No	No
COPD	No	No
Smoking	No	No
WBC, per microliter	17,600	10,500
Hb, g/dL	15.3	11.3
Platelets, 10^9^/L	193	319
AST, units/L	56	32
ALT, units/L	86	34
Total bilirubin, mg/dL	2.1	0.7
CRP, mg/L	18.3	
D-dimer, ng/mL	>5,000	>5,000
PT, seconds	NA	12.2
PTT, seconds	68.9	23.4
INR	NA	1.04
Ferritin, ng/mL	1,038.8	601.8
LDH, units/L	507	348
Troponins, ng/mL	0.33	0.26
BNP, ng/L	595	244
BMI, kg/m^2^	35.7	48.1

Case 2

A 63-year-old man with obstructive sleep apnea, chronic obstructive pulmonary disease on 2 L home oxygen, morbid obesity, heart failure, and recent diagnosis COVID-19 pneumonia (two weeks before presentation) complicated with an ischemic stroke presented with sudden onset shortness of breath, palpitation, and fatigue. On examination, heart rate was 112 beats per minute, blood pressure was 114/79 mm Hg, respiratory rate was 18 breaths/minute, and oxygen saturation was 96% on 2 L of oxygen. Respiratory and cardiovascular examinations were unremarkable. Neurological examination was significant for decreased motor strength to 3/5 bilaterally due to deconditioning and history of recent right basilar ischemic stroke.

**Figure 4:**
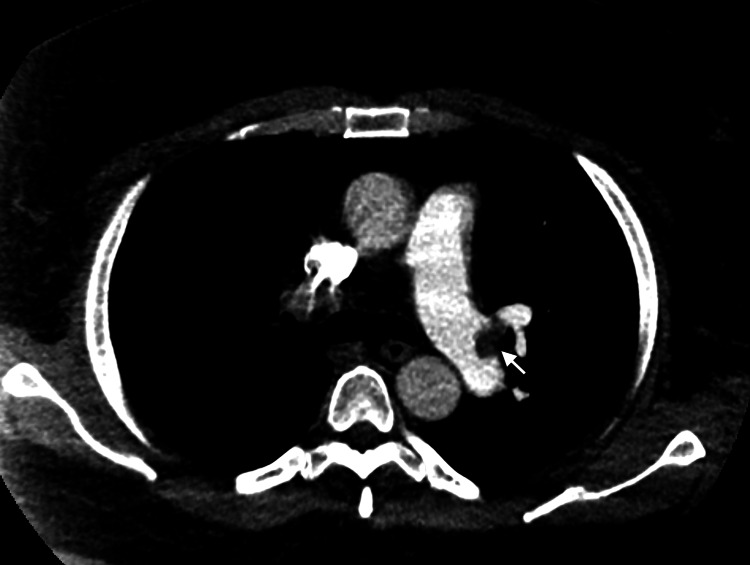
Axial view of a pulmonary CTA of the chest showing a small eccentric filling defect at the bifurcation of the left main pulmonary artery (marked by a white arrow) and extension into the left lower lobar artery. CTA, computed tomography angiogram

**Figure 5:**
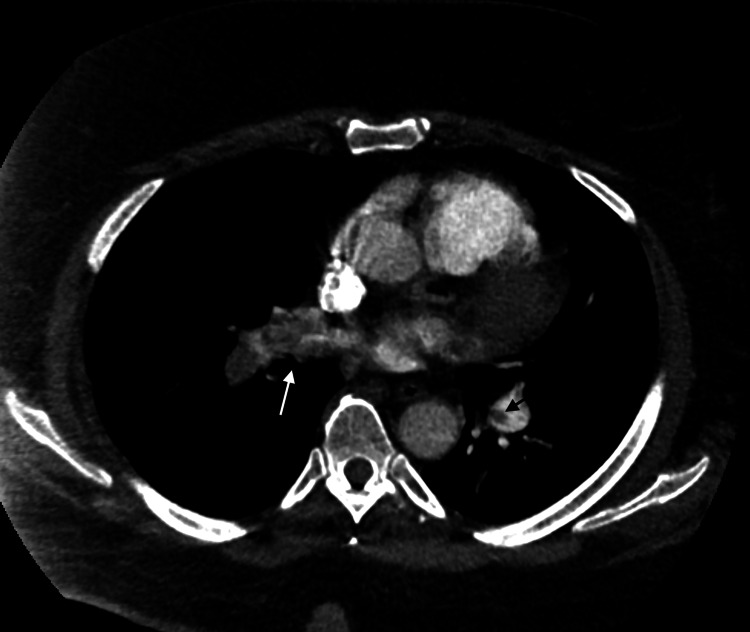
CTA of the chest on the right shows a large heterogeneous low attenuation structure (marked by a black arrow) in the right main pulmonary artery and right upper interlobar pulmonary artery consistent with having a moderate-to-large thrombus burden. An eccentric filling defect in the left pulmonary artery (marked by white arrow) is also seen. CTA, computed tomography angiogram

**Figure 6:**
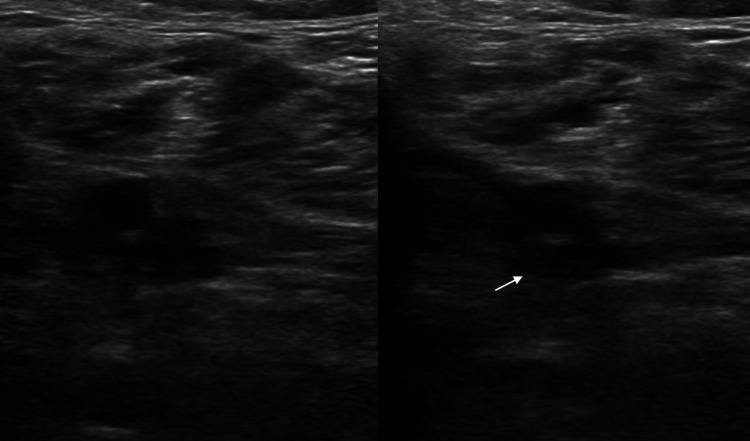
Duplex ultrasound of the right lower extremity. Transverse images of the right lower extremity veins show intraluminal echogenic material in the right posterior tibial artery, causing non-compressibility of the vessel (marked by a white arrow).

Laboratories workup on presentation is shown in Table 1. CTA chest revealed multiple bilateral acute pulmonary emboli with no evidence of right heart strain (Figures 4, 5). CT of the head was unremarkable. Vascular ultrasound of the bilateral lower extremities revealed near occlusive DVT of the right popliteal and posterior tibial veins with partially occlusive appearing thrombus (Figure 6). The left femoral vein, popliteal vein, and calf veins showed occlusive thrombus. Transthoracic echocardiogram showed evidence of mild right heart strain on visual assessment. The patient was started on IV heparin infusion and underwent catheter-directed thrombolysis. The patient was clinically improved over the course of hospital stay and discharge on oral anticoagulation to physical rehabilitation for post-stroke and deconditioning management.

## Discussion

In both the presented cases, the common thing is the D-dimer elevation, which put them at a higher risk of a PE. These are just two different PE presentations in patients with COVID-19 which tell us that we need to have a high clinical suspicion of a thrombotic event in a patient diagnosed with COVID. Timeline-wise, we do not know when a person is more at risk of developing a prothrombotic state. We suggest considering oral anticoagulation prophylaxis in the outpatient setting for patients with COVID symptoms with high baseline D-dimer levels to reduce life-threatening thromboembolism incidences. The retrospective study by Yin et al. showed that COVID-19 patients with high D-dimer levels on anticoagulation prophylaxis had a lower 28-day mortality compared to patients not on anticoagulation prophylaxis [5]. In a study by Paranjpe et al., the in-hospital mortality was lower in patients who received a therapeutic dose of anticoagulation versus prophylactic dose anticoagulation [6]. It comes with a caveat that anticoagulation increases the risk of bleeding in a patient and that it must be tailored to a specific patient need. Most importantly, we recommend not to start anticoagulation in patients who have a history of bleed or are at an increased risk of bleeding. A meta-analysis by McBane et al. including six retrospective studies on COVID-19 patients who received anticoagulation showed no significant mortality benefit in these patients [3]. COVID-19 is associated with an increased risk of VTE. These patients must be screened and treated to prevent life-threatening massive PE or other VTE complications. There is a need to develop stronger predictors to provide thromboprophylaxis in patients with COVID-19 disease to prevent life-threatening complications of VTE. COVID-19 patients should be suspected of increased risk of VTE. Symptomatic COVID-19 patients must be screened for VTE weekly with laboratory tests (D-dimer, procalcitonin, ferritin) and clinical symptoms. For COVID-19 patients with co-morbid conditions (diabetes mellitus, obesity, coronary artery disease) and reduced physical activity, outpatient thromboprophylaxis should be considered to reduce life-threatening VTE.

## Conclusions

COVID-19 is associated with an increased risk of VTE. These patients must be screened and treated to prevent life-threatening massive PE or other complications of VTE.
